# No outgrowth of chondrocytes from non-digested particulated articular cartilage embedded in commercially available fibrin matrix: an in vitro study

**DOI:** 10.1186/s13018-016-0355-4

**Published:** 2016-02-16

**Authors:** Nenad Andjelkov, Hans Hamberg, Per Bjellerup

**Affiliations:** Department of Orthopaedics, Västmanland County Hospital, Västerås, Sweden; Centre for Clinical Research, Uppsala University, Västmanland County Hospital, Västerås, Sweden; Department of Pathology, Västmanland County Hospital, Västerås, Sweden; Department of Clinical Chemistry, Västmanland County Hospital, Västerås, Sweden

**Keywords:** Articular chondrocytes, Particulated cartilage, Fibrin, Technique

## Abstract

**Background:**

Commercially available fibrin is routinely being used as both a matrix in certain cartilage repair techniques and a method for scaffold fixation. Chondrocytes from non-digested particulated cartilage fragments are proposed as a possible source for new cartilage tissue formation in some operative techniques. The goal of this study was to test that chondrocytes from particulated articular cartilage embedded in fibrin have an active role in the process of cartilage repair, as well as if commercially available fibrin should be used as a suitable matrix.

**Methods:**

Articular cartilage was obtained from patients undergoing total knee replacement surgery. The biopsies were particulated in small, 1–2-mm^3^ pieces and embedded in fibrin. Two groups were compared in our study, particulated articular cartilage with and without collagenase treatment. The specimens were analyzed by optical microscopy after 2–5 weeks of cultivation in a special construct embedded in a cell culture medium containing particulated cartilage embedded in fibrin in the upper phase and cancellous bone in the lower phase under the perforated nylon membrane.

**Results:**

None of the biopsies taken from four different patients showed the outgrowth of chondrocytes or bone marrow-originated cells into the fibrin matrix in our experimental model.

**Conclusions:**

It has been shown in our experimental model in vitro little to support the theory that articular chondrocytes from particulated articular cartilage embedded in fibrin have an active role in cartilage repair in its early stage.

## Background

A novel technique based on particulated cartilage embedded in a suitable carrier, fibrin matrix among others, has been recently introduced as an alternative treatment in cartilage repair. Basically, there are two different variants of the same technique: one using autologous articular cartilage fragments and the other cartilage fragments from juvenile allograft donor. These techniques are known as the Cartilage Autograft Implantation System (CAIS; DePuy/Mitek, Raynham, MA) and DeNovo Natural Tissue (NT; ISTO, St. Louis, MO) [1, 2].

The authors have reported that in laboratory and animal models, both techniques have shown the ability of transplanted cartilage cells to migrate from an extracellular matrix, divide, and form a new hyaline-like cartilage tissue matrix that integrates with the surrounding host tissue [2]. In another method for cartilage repair, namely, autologous chondrocyte implantation (ACI), it has been postulated that cartilage pieces have to be first treated with enzymes, i.e., collagenase, in order to “release” chondrocytes from the matrix, which can then migrate, multiply, and eventually create a new cartilage-like tissue formation [3].

The goal of this study was to test if chondrocytes are able to “escape” the cartilage without enzymatic digestion and, by doing so, may have an active role in new cartilage tissue formation, as well as if fibrin can be used as a suitable matrix in cartilage repair techniques.

## Methods

Cartilage and cancellous bone biopsies were obtained from four patients undergoing total knee replacement surgery. These patients had predominantly medial gonarthrosis, and consequently, a macroscopically normal cartilage, as judged by the surgeon, could be found in the lateral compartment of the knee. The cartilage biopsies were placed in a sterile 12-ml tube on 37 °C and in a matter of hours transferred to Dulbecco’s Modified Eagle’s Medium/Nutrient Mixture F-12 Ham (Cat. Nr. D8437, Sigma-Aldrich). Prior to that, amphotericin B and gentamicin were added to the medium in final concentrations of 2.5 and 50 μg/ml, respectively. The cartilage biopsies were minced to 1–2-mm^3^ pieces. Approximately half of this cartilage was transferred to another sterile 12-ml tube containing collagenase diluted in a cell culture medium to a total concentration of 0.8 mg/ml (collagenase type XI, C-9407, Sigma-Aldrich). The rest of the cartilage fragments were left in the tube with the cell culture medium alone, and both were then placed into a cell incubator (37 °C and 5 % CO_2_) overnight. The cancellous bone biopsies were placed in a sterile 24-well plate containing the cell culture medium with added antibiotics and then left in the cell incubator overnight.

**Fig. 1:**
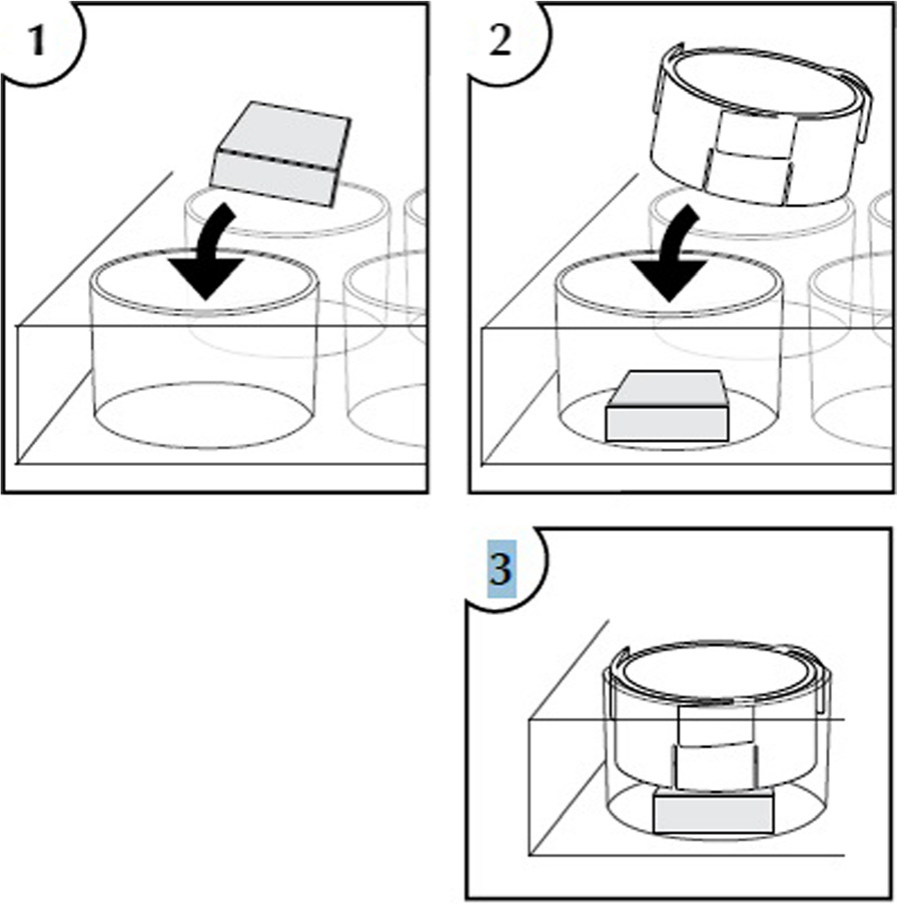
CellCrown™24 construct. With the courtesy of Scaffdex, Tampere, Finland

After 18–24 h of incubation, cartilage fragments digested in collagenase solution were washed two times by centrifuge at 1250 rpm and during 10 min. During the first wash, they were resuspended in a cell culture medium containing 10 % human sterile-filtered serum, second time without it. The final pellet was then placed in a special insert containing 8-μm PET or 1.2-μm nylon membrane (Fig. 1; CellCrown™24, Cat. Nr. C70001F, C12001F, Scaffdex, Tampere, Finland) perforated with a sterile 0.6 × 25-mm needle. The fibrin matrix (TISSEEL DUO QUICK, Baxter, Sweden) was then added covering in total cartilage fragments, and the inserts were placed onto the top of the cancellous bone biopsies previously situated in a sterile 24-well plate. The bone chips were prior to that resuspended in 1–1.5-ml growth medium containing 20 % human sterile-filtered serum. The whole construct was at last embedded in a growth medium by adding an extra medium onto the top of the insert. The cell culture medium was regularly changed, and the growth medium was replaced by a medium containing 10 % human sterile-filtered serum after 1 week of cultivation. After 2 weeks, the bone chips were removed from the wells. The inserts containing particulated cartilage embedded in fibrin were incubated during 2–5 weeks in total and then fixated in 4 % paraformaldehyde. Subsequently, they were embedded in paraffin and, when appropriate, ready to be analyze by optical microscopy. Eosin and trichrome staining were performed according to standard protocol as well as immunohistochemistry with S-100, a specific chondrocyte marker.

### Ethical approvals and informed consents

The Regional Ethical Review Board in Uppsala, at Uppsala Universitet has approved the study, and informed consent was obtained from all the patients donating their cartilage specimens for the study.

## Results

**Fig. 2:**
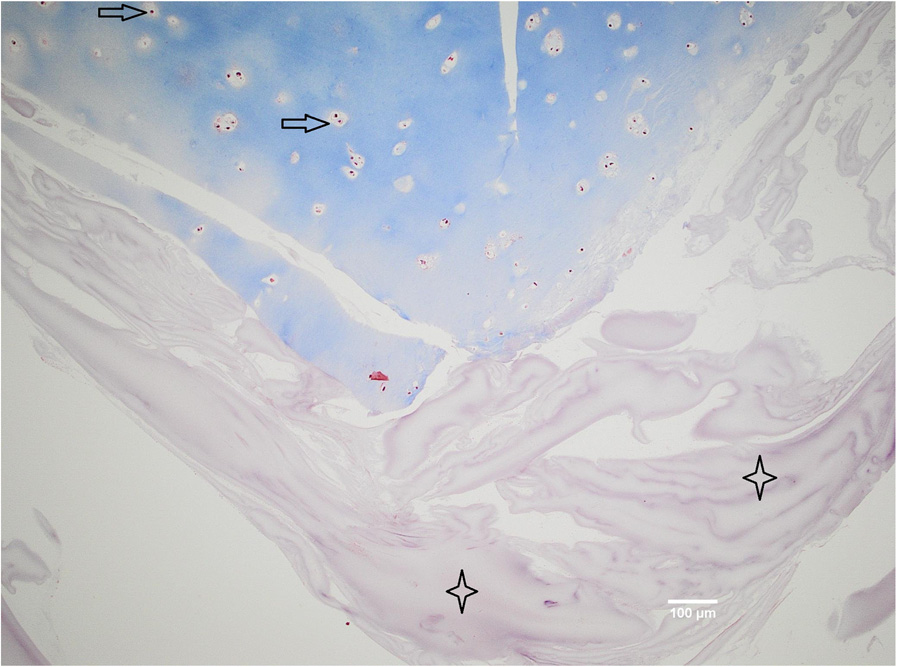
Particulated cartilage (in *blue*) embedded in fibrin matrix (in *violet*), ×10 magnification. Trichrome staining is not showing any presence of connective tissue in fibrin matrix (marked with *four-point stars*). Chondrocytes (marked with *arrow*) are located in the lacunas; there is no sign of cell migration into the matrix after 5 weeks of cultivation

**Fig. 3:**
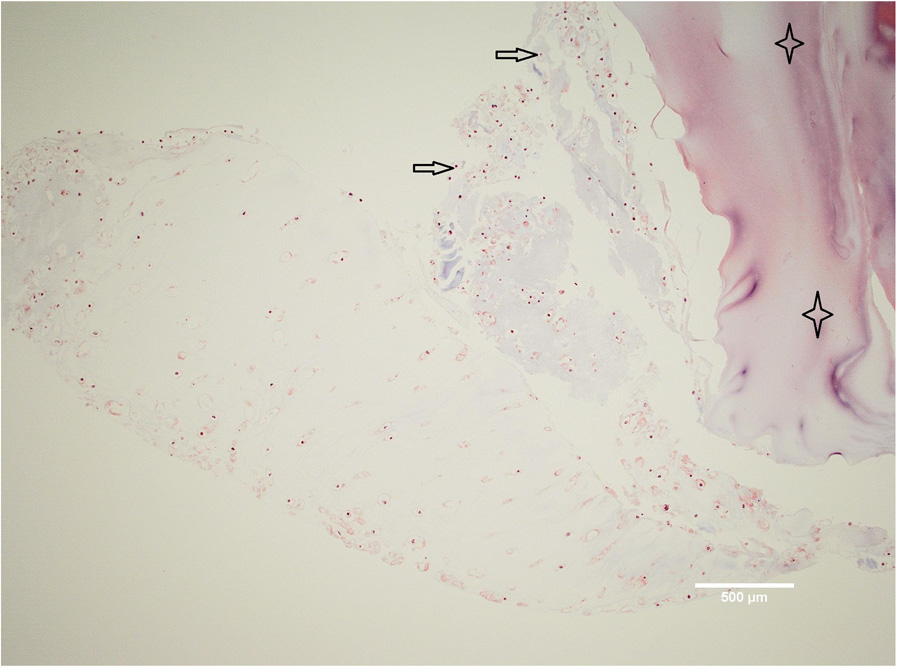
Particulated cartilage (*to the left* and *in the middle*) digested overnight with collagenase solution and thereafter embedded in fibrin matrix (*upper right corner*), ×10 magnification. Some of the cells (marked with *arrow*) have escaped cartilage matrix, but none of them have penetrated into the fibrin matrix (marked with *four-point stars*) after 5 weeks of cultivation. No staining for connective tissue with trichrome has been detected inside the fibrin matrix

**Fig. 4:**
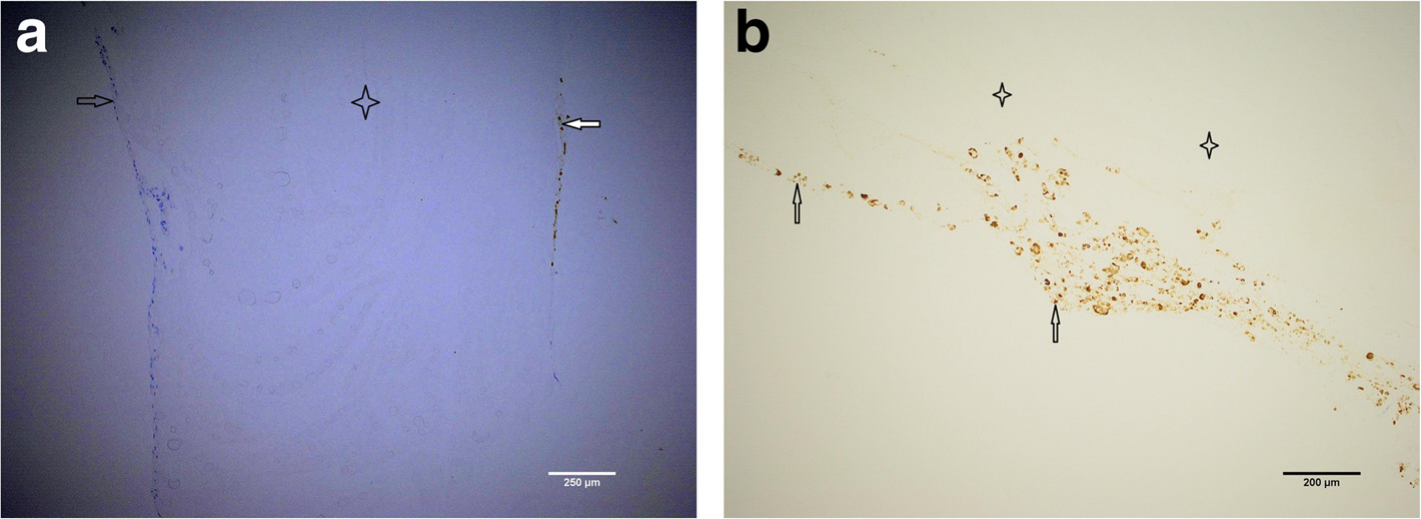
**a** Particulated cartilage fragments (stained in *brown* to the right with S-100) digested overnight with collagenase solution and thereafter embedded in fibrin matrix (*transparent* in the middle), ×10 magnification. Bone marrow-originated cells could be visible to the left stained in *blue*. These were lying at the upper phase of the perforated nylon membrane, which has been removed during the sample preparation. Both chondrocytes (marked with *white arrow*) and bone marrow-originated cells (marked with *black arrow*) have attached to the fibrin matrix (marked with *four-point star*) but have not penetrated it after 5 weeks of cultivation. **b** Particulated cartilage fragments digested overnight with collagenase solution and thereafter embedded in fibrin, ×10 magnification. Chondrocytes are stained (marked with *arrow*) with S-100 and have attached to the fibrin matrix (marked with *four-point stars*) but have not penetrated it after 5 weeks of cultivation

**Fig. 5:**
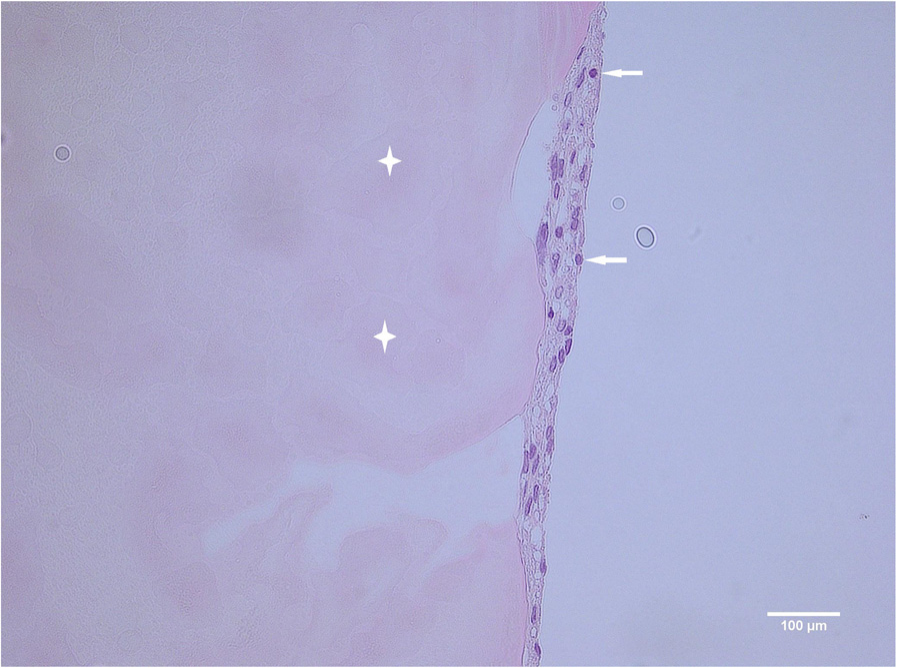
Particulated cartilage fragments digested overnight with collagenase solution and thereafter embedded in fibrin, eosin staining, ×40 magnification. Here, we can see bone marrow-originated cells (marked with *arrow*) in the lower phase, under the fibrin (marked with *four-point star*), and over the perforated nylon membrane (in this case, it would be found to the right from the cell layer), which has detached during the sample preparation. No cell invasion after 5 weeks of cultivation

We did not see the outgrowth of chondrocytes or penetration into the fibrin matrix in none of the biopsies taken from four different patients, not even after 5 weeks of cultivation (Fig. 2). In addition to that, the biopsies from samples treated with collagenase did show the outgrowth of chondrocytes but no invasion into the fibrin matrix (Fig. 3). The presence of chondrocytes was detected by S-100 (Fig. 4a, b). All other cells not stained with S-100 antibodies were assumed to have their origin from the bone marrow. The fibrin matrix showed poor integration with cartilage fragments (Fig. 2). No cell elements were noticed in the fibrin matrix, and therefore, no staining in fibrin for connective tissue with trichrome was found (Fig. 2). The cells that originated from the cancellous bone did migrate towards and through the membrane but did not penetrate further on into the fibrin matrix. On the contrary, they made a thin layer of cells on the upper side of the membrane and bellow the fibrin matrix (Fig. 5).

## Discussion

In our experimental model in vitro, we have seen the outgrowth of articular chondrocytes only in those specimens which have been digested with collagenase. The articular chondrocytes from the particulated, but non-digested, cartilage fragments have not shown any tendency for outgrowth. This finding is quite opposite to the claim of some other authors, namely, the establishers of the abovementioned novel operative technique/s [2]. According to these authors, the ability of articular chondrocytes to “escape” a cartilage has been proven in both laboratory and animal models [2]. The enzymatic digestion of the cartilage with collagenase has been postulated as absolutely necessary for the migration of articular chondrocytes out of the cartilage and their multiplication, as done in ACI [3]. A study showing goat articular chondrocyte outgrowth both in vitro and in vivo has been done [4]. The presence of chondrocyte outgrowth in vitro has been evident after 15 days and increased at 1 and 2 months. The cartilage fragments in this study have been embedded in fibrin and loaded onto a scaffold composed of a hyaluronic acid (HA)-derived membrane in the lower phase and platelet-rich fibrin matrix (PRFM) in the superior phase [4]. In our in vitro model, however, the human articular chondrocytes remained captured inside the fibrin matrix during the observed period of time, 2–5 weeks namely (Fig. 2). We have used neither HA-derived membrane nor PRFM, but still, it is a striking fact that we have not identified a single chondrocyte escaping the cartilage matrix. When digesting the cartilage fragments with collagenase overnight, the cells have escaped from the fragments, but a migration of cells into the fibrin matrix has not been observed either (Fig. 3). It has been suggested that a fibrin sealant promotes migration and proliferation of human articular chondrocytes in vitro [5]. On the other side, it has been reported that human fibrin glue hampered the healing process in rabbits in a similar model to that previously described in the text [4, 6]. The difference between the first named study [5] and our study is that we have used primary cartilage explants, non-digested as well, while primary chondrocyte culture has been used in the other case [5]. However, not even the cancellous bone-derived cells have penetrated the fibrin matrix, which speaks more in favor of fibrin-hampered chondrocyte migration rather than fibrin sealant promotion of cell migration as it has been described in the study mentioned above [5]. Human mesenchymal stem cells (hMSCs) have been used for the repair of osteochondral defects in rabbits by seeding them on biphasic composite constructs (hydroxyapatite + platelet-rich fibrin glue) for 4 and 8 weeks, respectively [7]. It has been postulated in this study that the group where differentiated hMSCs have been used has shown superior healing of osteochondral defects first after 8 weeks [7]. In vivo, fibrin will be gradually degraded during wound healing by fibrinolysis and replaced by the mature extracellular matrix, wherein the proteolytic activity of a membrane-type matrix metalloproteinases (MT1-MMP) [8, 9] and plasmin [10, 11] locally degrades the fibrin matrix. In vivo, this happens in a matter of days or weeks, and the rate of degradation depends on many factors such as fibrin structure, its cross-linking, and the incorporation of protease inhibitors [12–15]. Generally, the fibrin matrix is gradually replaced by mature collagen that is produced by invading cells [16–18]. In our study in vitro, there is a lack of such endogenous factors participating in the process of fibrinolysis, and one could therefore speculate that no invading cells have been found in the fibrin matrix. The insufficient degradation of fibrin and therefore scarce cell invasion could be the reason why no significant healing have been found after 4 weeks but first after 8 weeks as mentioned in the previously named study [5]. Fibrin is gradually degraded in vivo as it was already explained in the text, and the healing process succeeds its degradation, leading to the new tissue formation eventually. The initial inhibition of cell invasion and therefore new tissue formation that we have seen in our in vitro model have therefore probably no effect on the final clinical outcome. Indeed, the authors of this text have used routinely the fibrin matrix in our cartilage repair techniques and have seen neither poor defect fill nor poor clinical results that could be related to the use of the fibrin matrix [19, 20].

We have not seen as well in our in vitro study that articular chondrocytes from undigested cartilage fragments have the capability to escape from the cartilage matrix and, by doing so, have an active role in new tissue formation. Whether they can do that in an in vivo situation under the influence of, for example, endogenous collagenases [21, 22], we can just speculate. It seems that when implanting autologous chondrocytes using fibrin as a carrier, the results look fairly good [23]. On the other side, no tissue bonding or new cartilaginous tissue formation has been identified in the cartilage fragments without enzymatic treatment in a nude mice model [24]. Finally, two studies have shown no cell invasion into the fibrin matrix [25], as well as direct influence of the pore size and fibrin strand thickness on the cell invasion [26]. One recently done study has given even more evidence supporting our results in terms of chondrocyte outgrowth from adult human articular cartilage [27].

## Conclusions

In summary, the results of our in vitro study do not speak in favor of an early induction of cartilage defect repair by these techniques using undigested particulated cartilage fragments embedded in fibrin. However, their final outcome could be influenced by many endogenous factors that might be found in an in vivo situation. As a final conclusion, the use of a fibrin matrix does not seem to promote cartilage repair processes due to its initial inhibition of cell migration, and it seems that enzymatic digestion of particulated cartilage fragments is a prerequisite for the chondrocyte migration out of the cartilage matrix.
